# Papillary and Nonpapillary Calcium Oxalate Monohydrate Renal Calculi: Comparative Study of Etiologic Factors

**DOI:** 10.1100/tsw.2006.374

**Published:** 2006-04-18

**Authors:** Enrique Pieras, Antonia Costa-Bauz, Margarita Ramis, Felix Grases

**Affiliations:** ^1^ University Hospital Son Dureta, C/ Andrea Doria 55, E07014-Palma de Mallorca, Spain; ^2^ Laboratory of Renal Lithiasis Research, University Institute of Health Sciences Research, University of Illes Balears, Ctra. Valldemossa km 7.5, E07122-Palma de Mallorca, Spain

**Keywords:** calcium oxalate monohydrate, renal calculi, etiologic factors, papillary alteration, renal cavities

## Abstract

Calcium oxalate monohydrate (COM) renal calculi can be classified into two groups: papillary and nonpapillary. In this paper, a comparative study between etiologic factors of COM papillary and nonpapillary calculi is performed. The study included 40 patients with COM renal calculi. The urine of these individuals was analyzed. Case history, lifestyle, and dietetic habits were obtained.No significant differences between urinary biochemical data of both groups were observed; 50% of COM papillary stone formers and 40% of COM nonpapillary stone formers had urolithiasis family history. A low consumption of phytate-rich products was observed for both groups. A relationship between profession with occupational exposure to cytotoxic products and COM papillary renal lithiasis was detected.The results suggest that COM papillary calculi would be associated to papillary epithelium alterations together with a crystallization inhibitors deficit, whereas COM nonpapillary calculi would be associated to the presence of heterogeneous nucleants and a crystallization inhibitors deficit.

### INTRODUCTION

Calcium oxalate stones constitute more than 70% of urinary stones. Two different hydrates, the calcium oxalate monohydrate (COM) and the calcium oxalate dihydrate (COD), may be found in human stones[1]. It has been proved that the main etiologic factors for these groups are different. Thus, COM is observed to be more frequent in patients with normal urinary calcium excretions associated with a lack of crystallization inhibitory capacity, whereas COD is related with high levels of urinary calcium excretion and urinary pH $\geq 6$[2,3,4].

The COM calculi may be divided into two groups[5]: (1) COM papillary calculi, with a detectable attachment site to the papilla and basically consisting of a core near to the attachment site (concave zone), and radially striated concentrically laminated peripheral layers; and (2) COM calculi with no detectable site of attachment to papilla, developed in renal cavities, consisting of a central core that clearly serves as a nidus for stone body organization and development. Thus, the stone body is constituted by columnar COM crystals emerging from the core.

Several reports have been published since Randall's first description of papillary calcifications and their possible active role in the genesis of COM papillary calculi[6,7,8]. At present, it seems clear that renal epithelial cell injuries play a decisive role in such a type of renal calculi development[9,10,11,12,13].

On the other hand, the role of urinary biomolecules on COM crystal development has also been discussed. Thus, increase of calcium oxalate crystal nucleation and aggregation by peroxidized human proteins and renal cells has been postulated[14,15] and promotion by albumin and other urinary biomolecules on COM crystal formation has also been described[16,17], but no direct connections with the type of COM calculi formed (papillary or nonpapillary) have been established.

The aim of this paper is to present a comparative study between etiologic factors of COM papillary and nonpapillary calculi to understand better their mechanism of formation. COD stone formers are not studied because, at present, the differences between COM and COD stone formers are well established, being mainly related to hypercalciuria[2,3,4].

### PATIENTS AND METHODS

#### Patients

Our study included 40 patients with COM renal lithiasis (20 produced exclusively COM papillary calculi and 20 produced exclusively COM nonpapillary calculi). Both groups had the same sex and age distribution. The urine of these individuals was analyzed. Case history, lifestyle, and dietetic habits of these patients were obtained.

#### Renal Calculi Studies

Spontaneously passed renal stones were dried once collected, placed in sterile containers, and immediately studied. The procedure used to analyze and study renal calculi requires an appropriate combination of observation by means of macroscopic and microscopic conventional techniques (stereoscopic microscope; Optomic, Madrid, Spain) with physical techniques such as infrared spectrometry (Infrared Spectroscope Brucker IFS 66; Bruker, Ettlingen, Germany) and scanning electron microscopy (Hitachi S-530; Hitachi, Tokyo, Japan) coupled to X-ray microanalysis (Oxford Link Isis; Oxford, UK)[18].

The study of COM calculi (papillary or unattached) begins through the direct observation of its external aspect, using a stereoscopic microscope. Afterwards, each calculus is sectioned into two parts along a plane as near as possible to its geometric center, in order to be able to establish the internal structure and to identify the core of the calculus.

A typical papillary COM stone (see Fig. 1) basically consists of a excentrical core located near the concave region of the stone were it was attached to the papillae and a radially striated convex peripheral layer[5,19]. Scanning electron microscopic study permits detection of microcomponents present in the core and to confirm also the papillary origin of the calculus by observation of the concave external cavity. Thus, the presence of abundant organic matter and tubular apical cells demonstrates the presence of a point of attachment to the renal papillae.

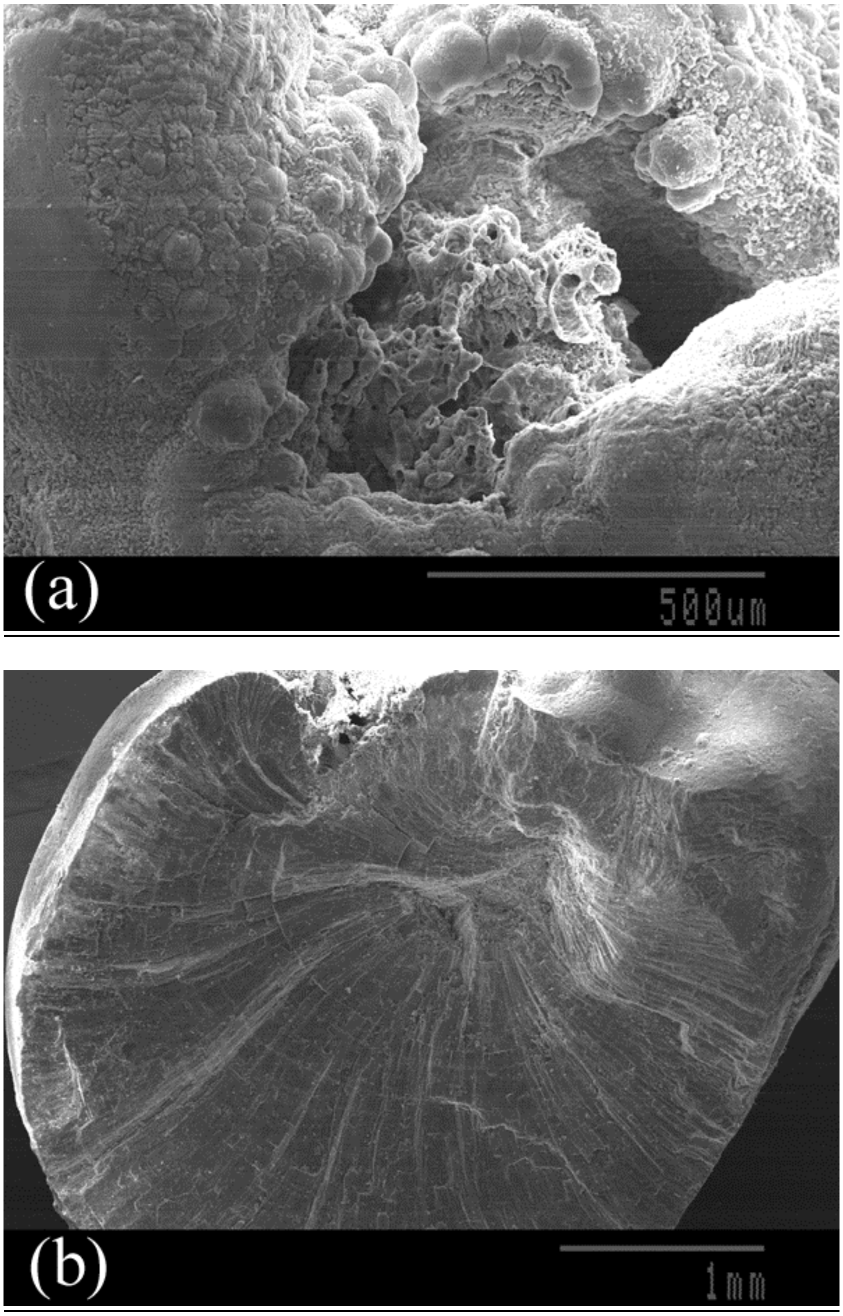

**FIGURE 1.** Scanning electron microscopy images of a COM papillary calculi. (a) Detail of the point of attachment to the renal papillae, in which calcified renal tubules can be observed; (b) general view of the section of the calculus in which the excentrical core located near the point of attachment to the papillae and a radially striated compact peripheral layer can be seen.

A typical unattached COM calculus (see Fig. 2) basically consists of a symmetrically round stone with a central core surrounded by columnar COM crystals emerging from the core and by the absolute absence of a site of stone attachment to the epithelium[5,19].

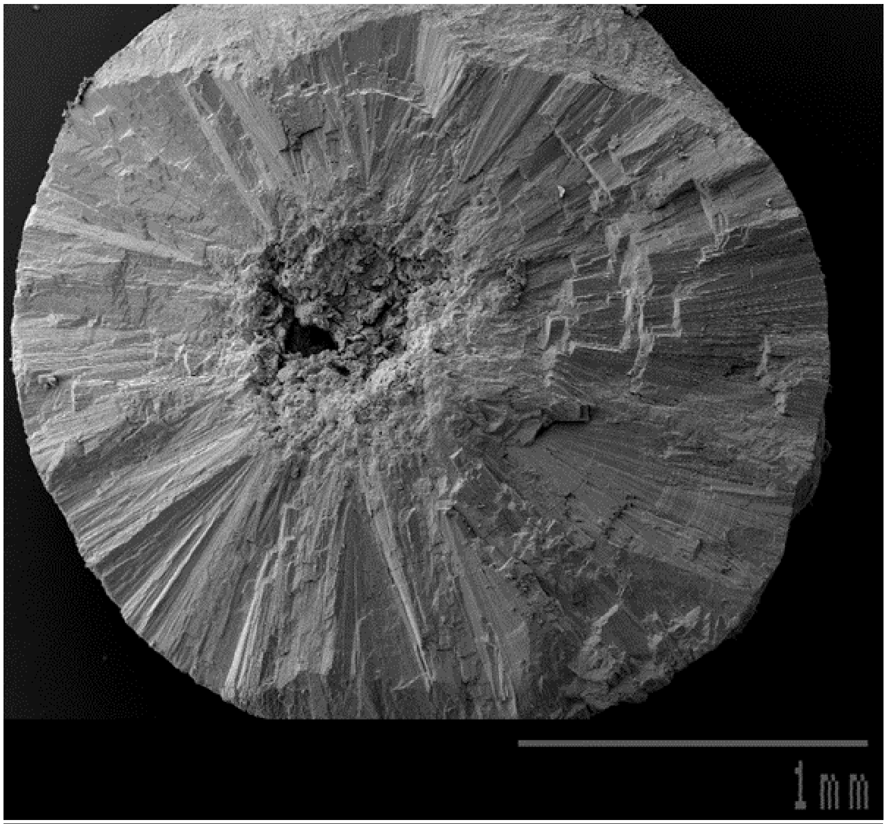

**FIGURE 2.** Scanning electron microscopy image of a COM nonpapillary calculi consisting of a central core surrounded by compact columnar COM crystals emerging from it.

### Analysis of Urinary Samples

All subjects were on free diet at the time of urine collection and none of the stone formers were undergoing pharmacological treatment of any kind. Serum analysis of these patients was performed (creatinine, calcium, magnesium, phosphorous, uric acid), mainly to evaluate the renal function. Patients with renal failure were excluded from this study. Subjects with infected urine were also excluded from the study. Twenty-four-hour urines were collected in sterile flasks containing thymol as a preservative, and immediately refrigerated. After collection, the volume was recorded and the samples stored at –20ºC until they were assayed. Two-hour urine collection was performed after overnight fasting and the pH was immediately measured with a glass electrode (Crison pH-meter). This urine was only used for pH evaluation since such measurement avoids pH changes due to precipitation processes (calcium salts) that can happen during 24-h storage. On the other hand, this pH measurement better represents the urinary basal pH since it is less affected by dietary factors. Normally urine was collected 1–2 months after stone passage/removal. Calcium, magnesium, and phosphorous were determined by inductively coupled plasma atomic spectroscopy. Uric acid and creatinine were determined by means of a Roche Modular Analytics with 11875426216 and 11875663216 reagents, respectively, and citrate and oxalate were determined by means of the R-Biopharm enzymatic test kits Nº 10139076035 and 10755699035, respectively. The urinary biochemical parameters were considered as potential lithogenic factors in abnormal conditions. Abnormal values for these parameters in accordance with our experience and the literature are defined in Table 1.

**TABLE 1**
**Urinary Metabolic Risk Reference Values in Urolithiasis**

| | |
|---|---|
| pH | < 5.5<br>> 6.0 |
| Creatinine (mmol/24 h) | > 17.7 |
| Ca (mmol/24 h) | > 6.2 (female)<br>> 7.5 (male) |
| Mg (mmol/24 h) | < 3.0 |
| P (mmol/24 h) | > 40.0 |
| Oxalate (mmol/24 h) | > 0.5 |
| Uric acid (mmol/24 h) | > 3.5 (female)<br>> 4.8 (male) |
| Citrate (mmol/24 h) | < 1.8 |

### Case History, Lifestyle, and Dietetic Habits of Patients

All patients were interviewed about urolithiasis family background, professional activity with special attention to those with possible exposure to cytotoxic compounds (pesticides, weed killers, disinfectants, cleaning products, fuels, etc.), and dietetic habits to be able to evaluate the phytate-rich products consumption (fiber, legumes, nuts, integral foods) through a 7-day food diary.

### Statistics

The chi-square ($\chi^2$) test was used to determine the differences between groups for the family background and exposure to cytotoxic products. Student's t test was used to determine the differences between groups in biochemical parameters and consumption of phytate-rich food. A probability of $p < 0.05$ was used for assessing statistical significance, unless otherwise specified.

## RESULTS

The urinary metabolic risk reference values in urolithiasis, used for comparative purposes, are shown in Table 1. The common urinary biochemical data (mean ± SEM) of both COM stone-former groups are shown in Table 2. As can be seen, no significant differences between them were observed and no potentially lithogenic parameters were detected (compare Tables 1 and 2). Nevertheless, observing the standard deviation values that appear in Table 2, it can be deduced that there were patients with hypocitraturia, hypercalciuria, hyperuricuria, or altered urinary pH.

The study of urolithiasis family background for both groups gave 50% of COM papillary stone formers with a family history of urolithiasis and 40% of COM nonpapillary stone formers with a family history. As can be observed, no differences between the two groups were detected.

From the study of dietetic habits, a low consumption of phytate-rich products (fiber, legumes, nuts, integral foods) was observed for both groups, but without differences between them (Table 3).

The study of the association between the exposure to cytotoxic products and the type of COM lithiasis shows 45% of COM papillary stone formers with professional activities related with occupational exposure to cytotoxic products and 25% of COM nonpapillary stone formers with professional activities related with occupational exposure to cytotoxic products. As can be seen, the obtained results, although not statistically significant, suggest some relationship between profession with occupational exposure to cytotoxic products and COM papillary renal lithiasis.

**TABLE 2**
**Urinary pH and Excretion Values of Different Urolithiasis-Related Biochemical Parameters (Values are Expressed as mean ± SD)**

| Urinary Parameter | COM Papillary Calculi Stone Formers (n = 20) | COM Nonpapillary Calculi Stone Formers (n = 20) |
|---|---|---|
| pH | 5.7 ± 0.6 | 5.8 ± 0.7 |
| Urinary volume (ml/24h) | 1750 ± 714 | 1473 ± 680 |
| Creatinine (mmol/24h) | 12.14 ± 3.93 | 10.43 ± 3.76 |
| Ca (mmol/24h) | 5.60 ± 4.60 | 5.15 ± 3.03 |
| Mg (mmol/24h) | 3.91 ± 1.73 | 3.46 ± 1.73 |
| P (mmol/24h) | 33.8 ± 18.2 | 26.5 ± 9.6 |
| Oxalate (mmol/24h) | 0.26 ± 0.10 | 0.22 ± 0.11 |
| Uric acid (mmol/24h) | 3.72 ± 1.38 | 3.46 ± 1.40 |
| Citrate (mmol/24h) | 2.87 ± 1.40 | 3.23 ± 1.51 |

**TABLE 3**
**Dietetic Habits Study: Consumption of Phytate-Rich Food (Legumes, Nuts, Dietetic Fiber, Wholemeal Breads and Other Wholegrain Products)**

| Consumption (times per week)* | Number of COM Papillary Calculi Stone Formers (n = 20) | Number of COM Nonpapillary Calculi Stone Formers (n = 20) |
|---|---|---|
| 0 | 0 | 1 |
| 2 | 4 | 3 |
| 4 | 7 | 6 |
| 6 | 4 | 7 |
| 8 | 3 | 3 |
| 10 | 2 | 0 |
| Mean consumption: | 5.2 | 4.8 |

* To obtain normal phytate levels, it was assumed to be necessary to consume phytate-rich food at least 7 times per week or more[21,22,23,24].

### DISCUSSION

As it was previously found[3,4], COM stone formers (papillary and nonpapillary) did not manifest clear alterations of the common biochemical urinary parameters and, in fact, they exhibited the same profile values as the healthy population. However, the high standard deviation values observed for citrate demonstrated that there were some patients with hypocitraturia. From the dietetic inquiry, a low consumption of phytate-containing products for the two groups of COM stone formers was observed. Phytate is a crystallization inhibitor whose presence in urine was clearly related to its dietary ingestion[20,21,22,23,24]. These facts support the hypothesis according to which COM lithiasis (papillary or nonpapillary) is associated to the crystallization inhibitor deficit.

Professional activities related with occupational exposure to cytotoxic products (inhalation of organic solvents, pesticides, disinfectants, etc.) seem to be related to COM papillary lithiasis. This could be justified by considering the cytotoxic effects of the mentioned products on papillary cells and epitheliums in general. The altered cells can induce COM development on their own surface[25,26], consequently originating a papillary calculus. In fact, it has been clearly demonstrated that the consumption of cytotoxic products like ethylene glycol or analgesic abuse induced the development of papillary calcifications[12,27].

The existence of familiar antecedents of renal lithiasis is similar and frequent for the two groups of COM stone formers. This can be explained assuming the importance of genetic factors, although different in the two groups. Thus, the existence of deep renal cavities can be a hereditary morphological factor that could be relevant in the formation of COM nonpapillary renal calculi, whereas the presence of sensitive epithelial mucosa with tendency to injury can be also a hereditary factor linked to COM papillary calculi. Obviously, genetic factors can be important, but they are not decisive since lithiasis is a multifactorial pathology and the development of a calculus also requires the participation of other important factors.

The results suggest that COM papillary calculi would be associated to papillary epithelium alterations together with a crystallization inhibitors deficit, whereas COM nonpapillary calculi would be associated to the presence of retained heterogeneous nucleants and crystallization inhibitors deficit.

### ACKNOWLEDGMENTS

Financial support by Govern de les Illes Balears, Conselleria d'Innovació i Energia, Direcció General d'R+D+I (PRDIB-2002GC1-04) and Ministerio de Ciencia y Tecnología, Dirección General de Investigación (BQU2003-01659) are gratefully acknowledged.

### BIOSKETCHES

**Enrique Pieras, MD, Ph.D.**, received his M.D. at Zaragoza University (Spain) in 1995, and a Ph.D. from University of Palma de Mallorca in 2004. Since 2002 he is template Urology Specialist at Universitary Hospital Son Dureta. His main research interest is urology, renal lithiasis and related topics. He colaborates in the research developped in the Laboratory of Renal Lithiasis Research. He has publised 8 scientific papers.

**Antonia Costa**, **Ph.D.,** received her B.Sc. Sciences (Chemistry) at Balearic Islands University, Palma de Mallorca (Spain) in 1988, and received a Ph.D. from Balearic Islands University in 1990. Since 1990 she has been Full Professor of the University of Balearic Islands. Her main research interest is renal lithiasis and related topics. She has carried out *in vitro* experiments using systems that emulate renal calculi formation. These studies together with the study of renal renal calculi permitted to clarify several aspects about the mechanism of calculi formation. She participated in the study of the properties of phytic acid as crystallization inhibitor and its usefulness in treatment of calcium oxalate renal lithiasis and other pathological calcifications (sialolithiasis, cardiovascular calcifications). She also colaborated in the development of a simple screening test to evaluate the urinary risk to develop calcium stones, that apart from diagnose purpose permits to evaluate the efficacy of a given therapy. She has publised 10 book chapters and about 80 scientific papers. She is also author of 8 patents.

**Margarita Ramis, PhD.,** received her B.Sc. Sciences (Chemistry) at Balearic Islands University, Palma de Mallorca (Spain) in 1987, and a Ph.D. from Balearic Islands University in 2001. Since 1989 she has been Secondary School Docent, and from 2004 she is Director of a Secondary School. Her main research interest is renal lithiasis and related topics. She colaborates in the research developped in the Laboratory of Renal Lithiasis Research. She has published 9 scientific papers.

**Felix Grases, PhD.,** received his B.Sc. Sciences (Chemistry) at Barcelona University, Barcelona (Spain) in 1974, and a Ph.D. from University of Palma de Mallorca in 1978. Since 1987 he has been Full Professor of the University of Balearic Islands and Director of the Chemistry Department from 1988 to 1995. At 1998, he founded the Laboratory of Renal Lithiasis Research at the Balearic Islands University, being the Director. His main research interest is renal lithiasis and related topics. He has carried out pioneering experiments using *in vitro* systems that emulate renal calculi formation. These studies together with the study of renal renal calculi permitted to clarify several aspects about the mechanism of calculi formation. He studied and demonstrated the properties of phytic acid as crystallization inhibitor and its usefulness in treatment of calcium oxalate renal lithiasis and other pathological calcifications (sialolithiasis, cardiovascular calcifications). He also developed a simple screening test to evaluate the urinary risk to develop calcium stones, that apart from diagnose purpose permits to evaluate the efficacy of a given therapy. He has publised numerous book chapters and about 250 scientific papers. He is also author of 9 patents.
